# Discoid Meniscus in Children: Why Meniscal Preservation and Repair Should Be the Goal in Modern Pediatric Knee Surgery

**DOI:** 10.7759/cureus.106296

**Published:** 2026-04-01

**Authors:** Javier Gonzalez Almonacid

**Affiliations:** 1 Orthopedics, Hospital Clinico San Borja Arriaran, Santiago, CHL; 2 Orthopedics, Clinica Alemana de Santiago, Santiago, CHL

**Keywords:** arthroscopic surgery, discoid meniscus, meniscal preservation, meniscal repair, pediatric knee surgery

## Abstract

Discoid meniscus is the most common congenital meniscal abnormality encountered in pediatric patients and frequently involves the lateral meniscus. While some discoid menisci remain asymptomatic, many children and adolescents develop pain, mechanical symptoms, or instability requiring surgical treatment. Historically, subtotal or total meniscectomy was commonly performed to address pain, mechanical symptoms, and functional limitation associated with symptomatic discoid meniscus. However, growing evidence regarding the biomechanical and long-term consequences of meniscal loss has led to a significant shift in treatment philosophy. Contemporary surgical management therefore emphasizes preservation of meniscal tissue whenever possible. Arthroscopic reshaping of the discoid meniscus combined with repair of associated tears or peripheral instability allows restoration of meniscal morphology while maintaining its biomechanical function. Given the long life expectancy and activity demands of pediatric patients, meniscal preservation should be considered the primary objective when surgically treating symptomatic discoid meniscus in children.

## Editorial

Discoid meniscus is the most common congenital meniscal abnormality encountered in pediatric knee surgery. The condition most frequently affects the lateral meniscus and is characterized by an abnormally thick and enlarged meniscal structure that covers a greater portion of the tibial plateau than a normal meniscus [1,2].

Although some discoid menisci remain asymptomatic, many children and adolescents present with pain, mechanical symptoms such as clicking or locking, snapping, or limited range of motion. The abnormal morphology predisposes the meniscus to tearing and instability, particularly during sports participation or low-energy trauma [3].

Historically, symptomatic discoid meniscus in children was frequently treated with subtotal or total meniscectomy. However, increasing understanding of meniscal biomechanics and long-term outcomes has significantly changed this approach. Modern surgical strategies now emphasize meniscal preservation and repair, particularly in the pediatric population [3].

For pediatric orthopedic surgeons, the question is no longer whether a symptomatic discoid meniscus should be treated, but how it should be treated while preserving as much meniscal tissue as possible.

Why the discoid meniscus is unique

The discoid meniscus differs from the normal meniscus not only in morphology but also as a developmental abnormality that may affect its structure and stability. Histologic studies have demonstrated altered collagen organization and increased thickness, factors that may contribute to the susceptibility of discoid menisci to tearing [2].

Another key characteristic is the frequent presence of peripheral meniscocapsular instability of the discoid meniscus. Detachment or laxity at the meniscocapsular junction may result in abnormal mobility of the meniscus and the classic “snapping knee” often described in children with discoid meniscus [1].

Because of these structural differences, discoid menisci are particularly prone to horizontal cleavage tears, complex tears, and peripheral detachments. These tear patterns are commonly encountered during arthroscopic evaluation of symptomatic children and adolescents [3].

Understanding these characteristics is essential when planning treatment. The objective should not simply be removal of abnormal tissue but restoration of a stable and functional meniscus.

Evolution of surgical treatment

For many years, subtotal or total meniscectomy was considered the standard treatment for symptomatic discoid meniscus. Early reports suggested that resection of the abnormal meniscus could relieve symptoms and restore knee function.

However, biomechanical studies demonstrated that removal of meniscal tissue significantly increases tibiofemoral contact pressures within the knee joint [4].

Clinical follow-up studies have also shown that meniscectomy performed at a young age is associated with an increased risk of early degenerative joint disease [5].

These findings have driven a paradigm shift in the management of discoid meniscus in children. Instead of removing the meniscus, contemporary surgical strategies focus on preserving as much functional meniscal tissue as possible while restoring stability and normal meniscal morphology.

Modern surgical concepts: saucerization and repair

Current surgical management of symptomatic discoid meniscus focuses on restoring a stable and functional meniscus while preserving as much native tissue as possible.

Arthroscopic reshaping of the discoid meniscus, commonly referred to as saucerization, aims to recreate a more physiologic semilunar configuration while maintaining an adequate peripheral rim. However, reshaping alone may not be sufficient in many cases.

Peripheral instability and associated meniscal tears are frequently present in symptomatic discoid meniscus and should be carefully assessed during arthroscopic evaluation. When instability or meniscocapsular detachment is identified, meniscal repair should be performed to restore stability and maintain the biomechanical function of the meniscus [3].

Several studies have reported favorable outcomes following saucerization combined with repair in pediatric patients. This combined approach allows preservation of functional meniscal tissue while restoring stability and biomechanics of the lateral compartment [3].

With advances in arthroscopic techniques and meniscal repair devices, surgeons are increasingly able to repair tear patterns that previously required partial meniscectomy. As a result, the concept of meniscal preservation in children has become central in modern pediatric knee surgery [1,2].

Why meniscal preservation matters in children

Meniscal preservation is particularly important in children and adolescents. The meniscus plays a crucial role in load distribution, shock absorption, joint stability, and lubrication within the knee joint.

As discussed previously, it is well established that removal of meniscal tissue increases tibiofemoral contact pressures and accelerates cartilage degeneration [4]. These biomechanical findings are consistent with long-term clinical studies demonstrating an association between meniscectomy and the development of early osteoarthritis, particularly when meniscal resection is performed at a young age [5].

Children with discoid meniscus have many decades of joint use ahead of them. For this reason, preservation of meniscal tissue should be prioritized whenever possible. Even complex tear patterns should be carefully evaluated for repair rather than immediate resection.

This meniscus-preserving philosophy has become a key principle in pediatric knee surgery. For pediatric orthopedic surgeons, this concept is particularly relevant when treating discoid meniscus, where the goal should not only be symptom relief but long-term preservation of joint health.

Clinical perspective

For the practicing pediatric orthopedic surgeon, the central principle when treating discoid meniscus in children is preservation of functional meniscal tissue. Saucerization alone may not be sufficient when peripheral instability is present. In these cases, meniscal repair should accompany reshaping procedures in order to maintain long-term joint biomechanics and reduce the risk of future degenerative changes.

Conclusion

The management of discoid meniscus in children has evolved significantly over recent decades. While meniscectomy was historically the standard treatment, growing evidence regarding the biomechanical and long-term consequences of meniscal loss has shifted surgical practice toward tissue preservation.

Arthroscopic reshaping combined with meniscal repair represents a widely accepted strategy for the treatment of symptomatic discoid meniscus. This approach restores more normal meniscal morphology while preserving the critical biomechanical functions of the meniscus.

In the era of modern arthroscopic techniques, meniscal preservation and repair should be considered the primary goal when treating symptomatic discoid meniscus in children.
